# A dataset of multi-contrast population-averaged brain MRI atlases of a Parkinson׳s disease cohort

**DOI:** 10.1016/j.dib.2017.04.013

**Published:** 2017-04-15

**Authors:** Yiming Xiao, Vladimir Fonov, M. Mallar Chakravarty, Silvain Beriault, Fahd Al Subaie, Abbas Sadikot, G. Bruce Pike, Gilles Bertrand, D. Louis Collins

**Affiliations:** a PERFORM Centre, Concordia University, Montreal, Canada; b McConnell Brain Imaging Centre, Montreal Neurological Institute, McGill University, Montreal, Canada; c Douglas Hospital Mental Health University Institute, McGill University, Montreal, Canada; d Division of Neurosurgery, McGill University, Montreal, Canada; e Department of Radiology, Hotchkiss Brain Institute, University of Calgary, Calgary, Canada

**Keywords:** Parkinson׳s disease, Atlas, Multi-contrast, MRI, Brain, Histology, Basal ganglia, Segmentation, T2*, Registration

## Abstract

Parkinson׳s disease (PD) is a neurodegenerative disease that primarily affects the motor functions of the patients. Research and surgical treatment of PD (e.g., deep brain stimulation) often require human brain atlases for structural identification or as references for anatomical normalization. However, two pitfalls exist for many current atlases used for PD. First, most atlases do not represent the disease-specific anatomy as they are based on healthy young subjects. Second, subcortical structures, such as the subthalamic nucleus (STN) used in deep brain stimulation procedures, are often not well visualized. The dataset described in this *Data in Brief* is a population-averaged atlas that was made with 3 T MRI scans of 25 PD patients, and contains 5 image contrasts: T1w (FLASH & MPRAGE), T2*w, T1–T2* fusion, phase, and an R2* map. While the T1w, T2*w, and T1–T2* fusion templates provide excellent anatomical details for both cortical and sub-cortical structures, the phase and R2* map contain bio-chemical features. Probabilistic tissue maps of whiter matter, grey matter, and cerebrospinal fluid are provided for the atlas. We also manually segmented eight subcortical structures: caudate nucleus, putamen, globus pallidus internus and externus (GPi & GPe), thalamus, STN, substantia nigra (SN), and the red nucleus (RN). Lastly, a co-registered histology-derived digitized atlas containing 123 anatomical structures is included. The dataset is made freely available at the MNI data repository accessible through the link http://nist.mni.mcgill.ca/?p=1209.

**Specifications Table**

**Table:** 

Subject area	*Neuroanatomy*
More specific subject area	*Brain MRI atlas*
Type of data	*Population-averaged brain MRI atlas & brain tissue segmentation*
How data was acquired	*3 T Magnetic resonance imaging*
Data format	*MINC1, MINC2 & NIFTI*
Experimental factors	*25 Parkinson׳s disease patients were scanned with a 10-echo multi-contrast FLASH and a T1w MPRAGE MRI sequence.*
Experimental features	T1–T2* fusion MRI was created for each patient, and used to drive group-wise registration to create a population-averaged multi-contrast atlas in the MNI ICBM152 space. Probabilistic tissue maps, manual segmentations of 8 subcortical nuclei, and a histology-derived digitized atlas of 123 anatomical structures are provided for the atlas.
Data source location	*Montreal, Canada*
Data accessibility	*The dataset is made available in public repository:*http://nist.mni.mcgill.ca/?p=1209

**Value of the data**

• Brain atlases of Parkinson׳s disease patients are currently rare.
• The publically available atlas represents the averaged anatomical and MRI intensity features of Parkinson׳s disease, with 5 different image contrasts.
• The T1–T2* fusion atlas conveniently visualizes cortical and subcortical structures in one image.
• Probabilistic brain tissue map, manual segmentation of eight subcortical nuclei, and a co-registered histology-based atlas are included.

## 1 Data

The dataset is a collection of six multi-contrast brain MRI atlases, accompanied by the associated probabilistic maps for three main brain tissue types, segmented labels for 8 subcortical nuclei, and a co-registered histology-based atlas. Derived from 3 T MRI scans of a cohort of 25 Parkinson׳s disease patients, the atlases were obtained by nonlinearly co-registering each patient׳s anatomy to a common space. The finished atlases are in MNI ICBM152 stereotactic space, with three image resolutions available: 1×1×1 mm^3^, 0.5×0.5×0.5 mm^3^, and 0.3×0.3×0.3 mm^3^.

## 2 Experimental design, materials and methods

### 2.1 Image acquisition

After informed consent, 25 Parkinson׳s disease patients (age=58±7 years, 13 male) were scanned with a T1w MPRAGE protocol and a multi-echo FLASH MRI protocol [1] on a Siemens 3 T Tim Trio MRI scanner. The whole-brain T1w MPRAGE had 176 sagittal slices (echo time (TE)=2.98 ms, repetition time (TR)=2300 ms, Inversion time (TI)=900 ms, flip angle (FA)=9°, BW=238 Hz/px, acquisition matrix=256×256, and resolution=1×1×1 mm^3^). The multi-contrast FLASH MRI contains 176 sagittal slices (TE={1.6, 4.1, 6.6, 9.1, 13.0, 16.0, 18.5, 21.0, 23.5, 26.0} ms, TR=30 ms, FA=23°, BW=±450 Hz/pix, acquisition matrix=256×256, resolution=0.95×0.95×0.95 mm^3^, 3/4 partial Fourier in the phase and slice encoding directions, and GRAPPA=2). From the multi-echo FLASH data acquired, four image contrasts were generated: T1w image, T2*w image, phase image, and R2* (i.e., 1/T2*) map. More specifically, the T1w and T2*w images are produced by averaging the magnitude images of the first four and last five echoes, respectively. The phase image is obtained via averaging the unwrapped phase images of the last five echoes using a homodyne filter, and the R2* map is computed by fitting all magnitude data to an exponential curve. As these contrasts are acquired simultaneously in one session, these processed images are inherently co-registered.

### 2.2 Image processing and atlasing

**Fig. 1:**
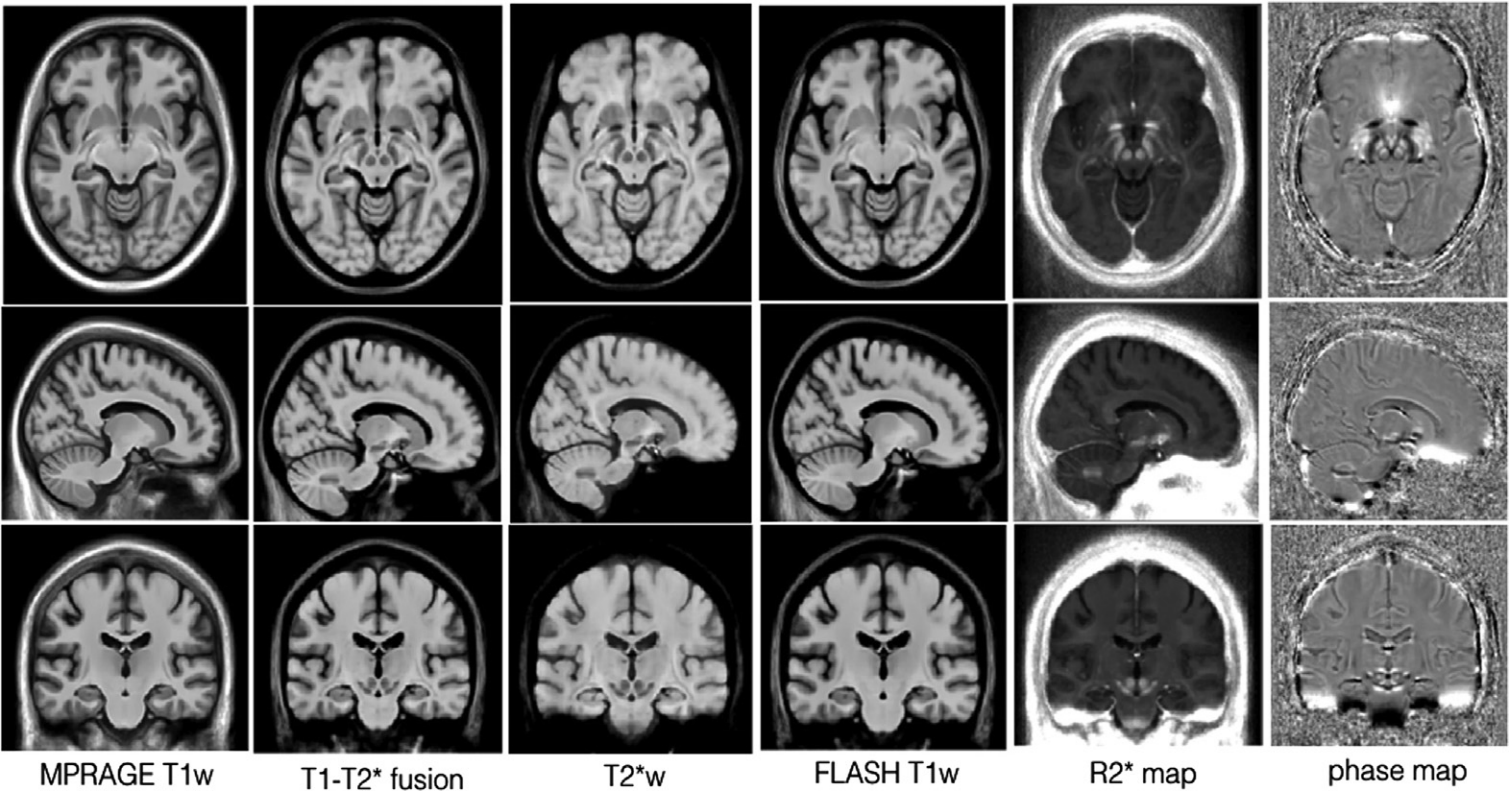
Multi-contrast population-averaged Parkinson׳s disease (1×1×1 mm^3^ resolution) atlases (columns from left to right: MPRAGE T1w, T1–T2* fusion, T2*w, FLASH T1w, R2* map, phase). The atlases are demonstrated with three slices: axial (Row 1), coronal (Row 2), and sagittal (Row 3). Note that these atlases include the population left-right asymmetry.

For each patient, the T1w MPRAGE MRI was rigidly registered to the multi-contrast FLASH dataset, and brain masks were generated using FSL brain extraction tool (BET) [2] based on the T1w MPRAGE MRIs. To facilitate inter-subject nonlinear registration, a T1–T2* fusion MRI was constructed for each individual using a spatially varying weighted combination of FLASH T1w and T2*w scans (full details in [3]). After non-local means denoising [4], non-uniformity correction [5], and intensity normalization, all T1–T2* fusion MRIs were first aligned to the MNI ICBM152 space [6] with full affine registration, and then all subject׳s data were co-registered together with an unbiased group-wise registration scheme [6]. Finally, the averaged result is the population-average T1–T2* fusion atlas, which combines cortical and subcortical details in one image, avoiding susceptibility artifacts in typical T2*w scans. Then, with the estimated deformation fields, atlases of other image contrasts (T1w (FLASH & MPRAGE), T2*w, phase, and R2* map) were created. For T1w (FLASH and MPRAGE) and T2*w contrasts, data were preprocessed in the same manner as the T1–T2* fusion images before atlas construction; for the phase image and R2* map, the data were used directly since their values do not require intensity standardization and should not be altered to truthfully reflect the biochemical properties of the tissue. As the appearances between the FLASH and MPRAGE T1w MRIs differ particularly for the cortex, due to different MRI acquisition methods, both contrasts were included in the dataset. Finally, three different image resolutions are provided for the atlases: 1×1×1 mm^3^, 0.5×0.5×0.5 mm^3^, and 0.3×0.3×0.3 mm^3^. While the first two resolutions cover the whole brain, the 0.3×0.3×0.3 mm^3^ resolution templates contain only the region for the subcortical nuclei. The atlases at 1×1×1 mm^3^ resolution are shown in Fig. 1.

### 2.3 Probabilistic tissue maps and anatomical annotations

**Fig. 2:**
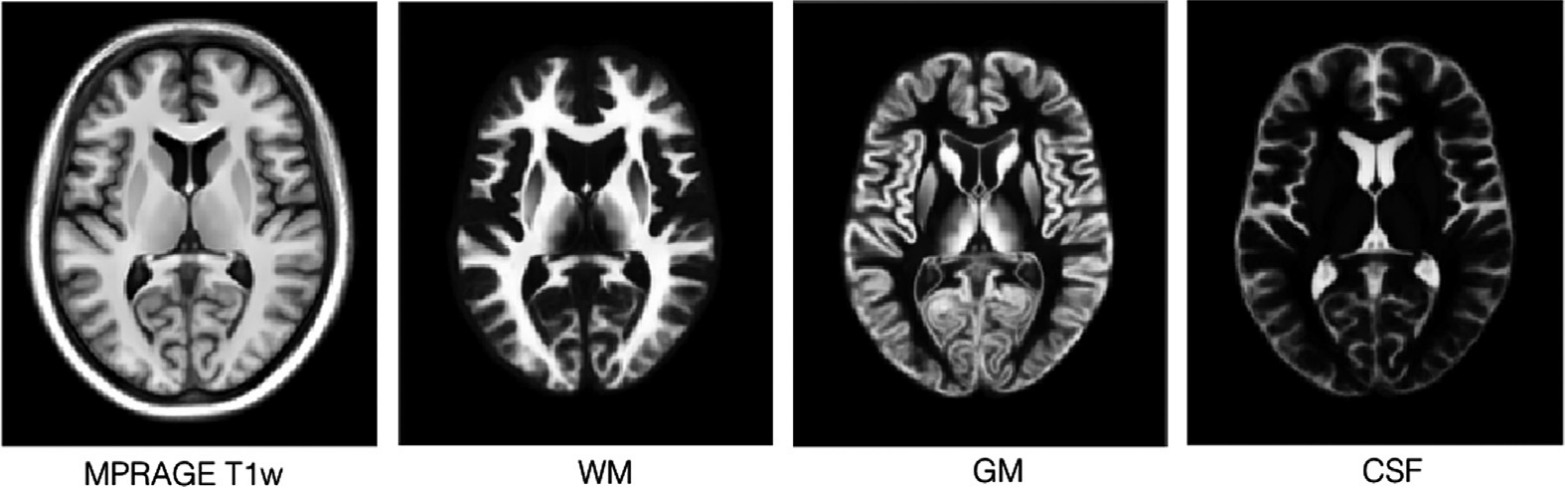
Probabilistic tissue maps for WM, GM and CSF with the corresponding view from the population-averaged MPRAGE T1w atlas. Note that due to partial volume effects, the boundary between CSF and WM appear bright in the GM map.

**Fig. 3:**
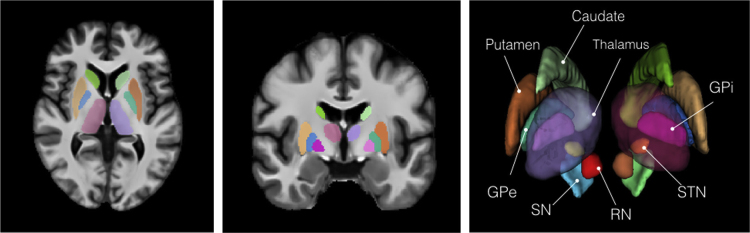
Segmented labels for the subcortical nuclei at 1×1×1 mm^3^ resolution. The labels are shown on the axial and coronal views of the population-averaged MPRAGE T1w atlas. A 3D rendering of the labels is also shown with annotations for each structure. To provide better visualization, the thalamus is rendered as semi-transparent.

**Fig. 4:**
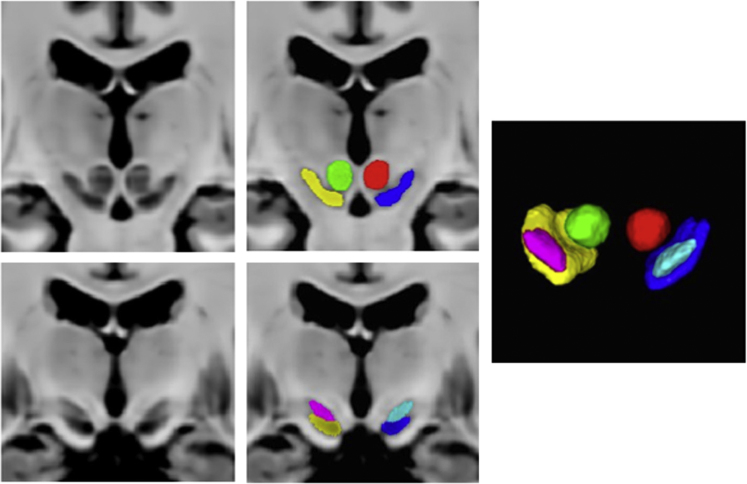
Segmented labels for the midbrain nuclei (SN, STN, and RN) at 0.3×0.3×0.3 mm^3^ resolution overlaid on the T1–T2* fusion atlas. The left and right STNs are shown as magenta and cyan labels, the left and right RNs are shown as green and red labels, and the left and right SNs are shown as yellow and blue labels.

**Fig. 5:**
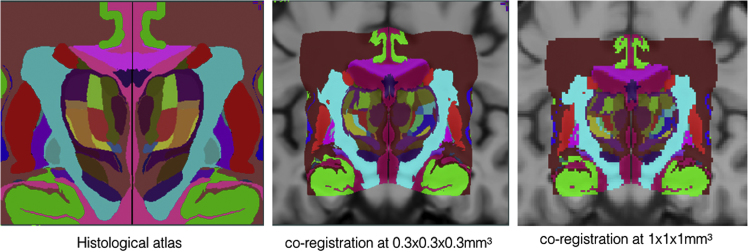
Co-registration of voxelized histological atlas [8] to the PD atlas at 0.3×0.3×0.3 mm^3^ resolution and 1×1×1 mm^3^ resolution.

Three sets of tissue segmentations are provided: probabilistic tissue maps for white matter (WM), grey matter (GM) and cerebrospinal fluid (CSF), segmented labels for subcortical nuclei at 1×1×1 mm^3^ resolution, and segmented labels for midbrain nuclei at 0.3×0.3×0.3 mm^3^ resolution. First, fuzzy segmentations of WM, GM and CSF were obtained using a minimum distance classifier [7] for each patient, and the classification results are in the range of [0,1] roughly representing partial volume effects for each tissue class. The probabilistic tissue maps were generated by averaging the deformed individual fuzzy tissue segmentations in the population template space with the deformation fields previously generated. The results are shown in Fig. 2. Second, eight subcortical nuclei were manually segmented bilaterally in 3D at 1×1×1 mm^3^ resolution using ITK-SNAP (http://www.itksnap.org). More specifically, the caudate nucleus, the putamen, and the thalamus were segmented using T1w MPRAGE atlas. The globus pallidus internus and externus (GPi & GPe) were segmented based on the T1w MPRAGE and T2*w atlas, and the subthalamic nucleus (STN), the substantia nigra (SN), and the red nucleus (RN) were segmented with the T2*w atlas. The segmentation results are shown in Fig. 3. Lastly, to provide better delineation for nuclei with smaller volumes, the STN, SN and RN were manually re-segmented based on the T2*w atlas at 0.3×0.3×0.3 mm^3^ resolution (see Fig. 4). The label numbers and the associated structures for both sets of segmentations are detailed in Table 1. In addition, the Gilles-Mallar atlas [8] derived from histological data was nonlinearly registered to the population-averaged template (Please refer to [3] for more details). The atlas contains 123 structures, and the co-registered atlas at the resolution of 0.3×0.3×0.3 mm^3^ and 1×1×1 mm^3^ (see Fig. 5) are provided within the dataset. The label numbers with the corresponding anatomical structures defined in Schaltenbrand and Wahren [9], Gloor [10], and Hirai and Jones atlases [11] are listed in Table 2. Please note that we do not recommend using the basal ganglia-thalamic atlas, as is, as a clinical tool. Rather, we strongly recommend that each user undertake their own validation and diligence prior to use of the atlas for any research or clinical purpose in human.

## Figures and Tables

**Table 1:** Label numbers for the subcortical nuclei segmentations with the corresponding structures. Note that the label numbers are consistent between the segmentations at 1×1×1 mm^3^ and 0.3×0.3×0.3 mm^3^.

Label number	Nuclei	Label number	Nuclei
1	Left red nucleus	2	Right red nucleus
3	Left substantia nigra	4	Right Substantia nigra
5	Left subthalamic nucleus	6	Right subthalamic nucleus
7	Left caudate	8	Right caudate
9	Left putamen	10	Right putamen
11	Left globus pallidus externa	12	Right globus pallidus externa
13	Left globus pallidus interna	14	Right globus pallidus interna
15	Left thalamus	16	Right thalamus

**Table 2:** Label numbers and anatomical structures in the co-registered histological atlas.

Label	Schaltenbrand and Wahren	Gloor	Hirai and Jones	Notes	Label	Schaltenbrand and Wahren	Gloor	Hirai and Jones	Notes
1	Striatum				2	Cortex			
3	Claustrum				4	Internal capsule			
5	Globus Pallidus (Pm)				6	Nucleus amygdala profundus lateralis (A. p. l.)	Lateral nucleus (L)		Amygdala
7	Optic Tract (II)				8	Nucleus amygdala profundus intermedius (A. p. l.)	Basal nucleus (B)		Amygdala
9	Anterior commissure (Cm.a.)				10	Lateral medullary lamina (la.p.l)			
11	Globus Pallidus Internal (Pm.I)				12	Globus Pallidus external (Pm.e)			
13	Anterior Perforated substance (B)				14	Nucleus amygdalae profundus lateralis (A.p.m)	Accessory Basal Nucleus (AB)		Amygdala
15	Ventro-oralis internus (V.o.i.)			Thalamus	16	Stratum septi pellucidi (Str.sep)			
17	Pro-thalamicus principalis centralis (Pth. Pr. Ce.)	Bed nucleus of the stria terminalis (BNST)		Hypothalamus	19	Nucleus facialis (VII)			
20	Nucleus amygdalae profundus ventralis (A.p.v)	Para Laminar nucleus (PL)		amygdala	21	Medial medullary lamina (la.p.m)			
22	Stria medullaris thalami (st. m)				23	Nucleus paraventricularis hypothalamic (Pv)			
24	Nucleus Reticulatus Polaris (Rt.po.)				25	Zona incerta (Z.i.)			
26	Nucleus lateropolaris thalami (Lpo)		Ventral Anterior Nucleus(VA)	Thalamus: see lables 36,89,90	27	Nucleus fasciculosus thalami (Fa)		Medioventral Nucleus (MV)	Thalamus
28	Nucleus Anterior Principalis (Apr)		Antero-ventral nucleus	thalamus	29	Mamillary body (M.m)			
35	Fornix (Fx)				36	Dorso-oralis externus (D.o.e)		Ventral Anterior Nucleus (VA)	Thalamus: see labels 26,89,90 for HJ thalamus
37	Nucleus Medialis (M)		Mediodorsal Nucleus (MD)	Thalamus	39	Subthalamic nucleus (Sth)			
40	Lamella medialis thalami (La. M.)			Thalamus	41	Campus Forellii (pars H2)			Thalamus
47	Pars compacta (Ni.c)/pars reticula (Ni.r)			Substantia nigra	48	Ruber (Ru)			Red nucleus
49	Nucleus Centralis (Ce.)		Central Median Nucleus (CM)	Thalamus	51	Nucleus Parafasiculairs (Pf.)			Thalamus
52				WM in red nucleus and travelling towards the thalamus	53	Nucleus Dorsalis superficialis (D.sf.)		Lateral dorsal nucleus (LD)	Thalamus
60	Fasciculus gracillis Goll (G)				61	Paegeniculatum (prG)			
63	Penduncle				64	Nucleus peripendicularis (Ppd.)			Thalamus
66	Ganglion habenulae medialis (H.m)			Forms Hb with 67	67	Ganglion habenulae internus (H.i)			Forms Hb with66
68	Corpus geniculatum mediale (G.m/G.Md)				70	Nucleus Limitans (Li)		Nucleus Limitans (Li)	Thalamus
71	Ventro-caudalis parvocell (V.c.pc)		Basal nucleus Medial nucleus/Ventral posterior inferior nucleus (VMb/VPI)	Thalamus	73	Lemniscus medialis (L.m)			
74	Brachium colliculi inferioris (B.co.i)				75	Nucleus Vestibularis (VIII)			
76	Area trangularis Wernicke (A.tr.W)				81	Ventro-oralis medialis (V.o.m.)		Ventral Medial Nucleus (VM)	Thalamus
86	Ventro-oralis internus (V.o.i.)		Ventral lateral posterior nucleus (VLp)	Thalamus: see labels 86,91,92,93,94,104,111,112,114,120 for HJ	87	Ventro-oralis anterior (V.o.a.)		Ventral lateral anterior nucleus (VLa)	Thalamus: see labels 87,88,91,123 for HJ
88	Ventro-oralis posterior (V.o.p.)		Ventral lateral anterior nucleus (VLa)	Thalamus: see labels 87,88,91,123 for HJ	89	Dorso-oralis internus (D.o.i)		Ventral anterior nucleus (VA)	Thalamus: see labels 26,36,90 for HJ
90	Zentro-lateralis oralis (Z.o.)		Ventral lateral anterior nucleus (VLa)	Thalamus: see labels 87,88,91,123 for HJ	91	Ventro-intermedius internus (V.im.i)		Ventral lateral posterior nucleus (VLp)	Thalamus: see labels 86,91,92,93,94,104,111,112,114,120 for HJ
92	Zentro-lateralis externus (Z.im.e)		Ventral lateral posterior nucleus (VLp)	Thalamus: see labels 86,91,92,93,94,104,111,112,114,120 for HJ	93	Zentro-intermedius internus (Z.im.i)		Ventral lateral posterior nucleus (VLp)	Thalamus: see labels 86,91,92,93,94,104,111,112,114,120 for HJ
94	Ventro-intermedius externus (V.im.e)		Ventral lateral posterior nucleus (VLp)	Thalamus: see labels 86,91,92,93,94,104,111,112,114,120 for HJ	95	Ventro-caudalis internus (V.c.i)		Ventral posterior medial nucleus (VPM)	Thalamus: see labels 95,113
96	Ventro-caudalis anterior internus (V.c.a.e)		Ventral posterior lateral nucleus (VPLa)	Thalamus: see labels 96,97,98 for HJ	97	Zentro caudalis externis (Z.c.e)		Ventral posterior lateral nucleus (VPLa)	Thalamus: see labels 96,97,98 for HJ
98	Zentro caudalis internis (Z.c.i)		Ventral posterior lateral nucleus (VPLa)	Thalamus: see labels 96,97,98 for HJ	99	Dorso-caudalis (D.c.)		Lateral posterior nucleus (LP)	Thalamus: see labels 99,100,101
100	Nucleus pulvinaris orolateralis (Pu.o.l)		Lateral posterior nucleus (LP)	Thalamus: see labels 99,100,101	101	Nucleus pulvinaris oromedialis (Pu.o.m.)		Lateral posterior nucleus (LP)	Thalamus: see labels 99,100,101
102	Ventro-caualis portae (V.c.por)		Anterior pulvinal nucleus (Pla)	Thalamus: see labels 102,103	103	Nucleus pulvinaris oroventralis (Pu.o.v)		Anterior pulvinal nucleus (Pla)	Thalamus: see labels 102,103
104	Nucleus ventroimtermedius internus (V.im.i)		Posterior Nucleus (VLp)	Thalamus: see labels 86,91,92,93,94,104,111,112,114,120 for HJ	105	Nucleus pulvinaris intereniculatus (Pu.ig)		Inferior pulvinar nucleus (Pli)	Thalamus
106	Nucleus pulvinaris (Pu.m)		Medial pulvinar nucleus (Plm)	Thalamus	107	Pulvinar laterale (Pu.l)		Lateral pulvinar nucleus (Pll)	Thalamus
108	Corpus collsum				109	Cerebro-spinal fluid			
110	General white matter				111	Dorso-intermedius internus (D.im.i)		Ventral lateral posterior nucleus (VLp)	Thalamus: see labels 86,91,92,93,94,104,111,112,114,120 for HJ
112	Dorso-intermedius externus (D.im.e)		Ventral lateral posterior nucleus (VLp)	Thalamus: see labels 86,91,92,93,94,104,111,112,114,120 for HJ	113	Ventro-caudalis anterior internus (V.c.a.i)		Ventral posterior medial nucleus (VPM)	Thalamus: see label 95,113
114	Zentro-lateralis intermedius (Z.im)		Ventral lateral posterior nucleus (VLp)	Thalamus	115	Ventro-caudalis externus (v.c.e)		Ventral posterior lateral nucleus (VPLa)	Thalamus: see labels 115,118
116	Nucleus pulvinaris superficialis (Pu.sf)			Thalamus	117	Ventro-caudalis parvocell externus (V.c.pc.e)		Ventral posterior inferior nucleus (VPI)	Thalamus
118	Ventro-caudalis posterior externus (V.c.p.e)		Ventral posterior lateral nucleus (VPLa)	Thalamus	119	Pulvinar mediale (Pu.m)		Medial pulvinar nucleus (Plm)	Thalamus
120	Ventro-oralis posterior (V.o.p)		Ventral lateral posterior nucleus (VLp)	Thalamus: see labels 86,104,120 for HJ	121	Zentro-intermedius externus (Z.im.e)		Ventral lateral anterior nucleus (VLa)	Thalamus
122	Ventro-intermedius externus (V.im.e)		Ventral lateral anterior nucleus (VLa)	Thalamus	123	Dorso-oralis internus (D.o.i)		Ventral lateral anterior nucleus (VLa)	Thalamus: see labels 87,88,91,123 for HJ
