# Supplementary material for: A dataset of multi-contrast population-averaged brain MRI atlases of a Parkinson׳s disease cohort
Source: Data Brief. 2017 Apr 15;12:370–9. doi: 10.1016/j.dib.2017.04.013 (PMC5413210; doi:10.1016/j.dib.2017.04.013)
Supplement: Supplementary file 1 — Supplementary material [file mmc1.docx]

No authors of this article are members of Japanese Surgical Society (JSS). All authors have no conflict of interests to declare.
